# Supplementary material for: Complexome profiling of the Chlamydomonas psb28 mutant reveals TEF5 as an early PSII assembly factor
Source: Plant Cell. 2025 Mar 17;37(6):koaf055. doi: 10.1093/plcell/koaf055 (PMC12164590; doi:10.1093/plcell/koaf055)
Supplement: koaf055_Supplementary_Data [file koaf055_supplementary_data.zip › psb28_interactive.zip!/plots/intensity/qualityPlots/peptides/LHCA1_Cre06.g283050.t1.2.html]

### Cre06.g283050.t1.2

Trivial name: LHCA1  
SeqCoverage [median]: 45.20  
Mass: 23.90 kDa  
  
 

| Sequence | MissedCleavage | Mass [Da] | wtIntensity | muIntensity | Score |
| --- | --- | --- | --- | --- | --- |
| AGNWLPGSDAPAWLPDDLPGNYGFDPLSLGKEPASLKR | 2 | 4021.00 | 15018.40 | 0.00 | 39.11 |
| DSSKSGELKLK | 2 | 1190.65 | 1192.20 | 3963.15 | 98.11 |
| EIKNGRLAMVAFLGFVAQHAATGK | 2 | 2528.37 | 1660.11 | 0.00 | 52.32 |
| EPASLKR | 1 | 799.46 | 124155.02 | 90581.18 | 183.89 |
| FTESEVIHGR | 0 | 1173.58 | 3201588.93 | 2645620.99 | 170.85 |
| GDAGGVVYPGGAFDPLGFAK | 0 | 1893.93 | 984551.00 | 415893.56 | 203.85 |
| GDAGGVVYPGGAFDPLGFAKDSSK | 1 | 2311.11 | 31026.83 | 18033.80 | 109.05 |
| LAMVAFLGFVAQHAATGK | 0 | 1830.98 | 1457691.76 | 1524980.75 | 198.95 |
| LKEIKNGRLAMVAFLGFVAQHAATGK | 3 | 2769.55 | 7980.57 | 0.00 | 89.71 |
| NGRLAMVAFLGFVAQHAATGK | 1 | 2158.15 | 8351.37 | 0.00 | 110.56 |
| RFTESEVIHGR | 1 | 1329.68 | 173545.32 | 147849.02 | 168.71 |
| SGELKLK | 1 | 773.46 | 799.68 | 16569.97 | 143.00 |
| SGELKLKEIK | 2 | 1143.69 | 3199.44 | 2060.13 | 79.82 |
|  |  |  | 6010760.6 | 4865552.5 |  |
